# Radiological Correlates of Headache and Papilledema in Patients Undergoing Cranial MRI: Clinical Relevance of the Evans Index and Fazekas Score

**DOI:** 10.7759/cureus.116731

**Published:** 2026-09-22

**Authors:** Abdulkadir Eren, Emrah Karatay, Rabia Mercan Sahin, Hasan Sermet

**Affiliations:** 1 Radiology, Istanbul Medipol University Mega Hospital, Istanbul, TUR; 2 Radiology, Sultan II Abdulhamid Han Training and Research Hospital, Istanbul, TUR; 3 Radiology, Famagusta State Hospital, Famagusta, CYP

**Keywords:** evans index, fazekas score, headache, intracranial hypertension, papilledema

## Abstract

Background/Aim: The Evans index and Fazekas score are among the most frequently reported incidental findings on cranial magnetic resonance imaging (MRI), yet their relationship with confirmed headache and papilledema is not well characterized outside the normal pressure hydrocephalus (NPH) literature. In this exploratory, hypothesis-generating study, we evaluated this association in patients undergoing cranial MRI for headache and/or visual disturbance.

Materials and methods: In this retrospective study, cranial MRI of 224 patients (two non-overlapping cohorts, n=100 and n=124) was evaluated by one of two radiologists, who graded the Fazekas score and Evans index. Headache severity (0-3) and papilledema (present/absent) were obtained from clinical records. Associations were assessed with chi-square, Mann-Whitney U, Spearman correlation, and multivariable logistic regression adjusted for age and sex; a sensitivity analysis additionally adjusted for the collinear reader/cohort variable.

Results: Papilledema was present in 47 patients (21.0%). The Evans index was independently associated with papilledema (adjusted OR 2.07/0.05-unit increase, 95% CI 1.27-3.33, p=0.004) and headache (adjusted OR 1.52, 95% CI 1.01-2.25, p=0.047); the Fazekas score showed no independent association with either. The papilledema association was non-significant on univariate testing (p=0.206). After adjusting for reader/cohort, the papilledema association strengthened (adjusted OR 2.76, 95% CI 1.59-4.80, p<0.001), while the headache association was attenuated (adjusted OR 1.37, 95% CI 0.89-2.09, p=0.148).

Conclusion: The Evans index, but not the Fazekas score, was associated with papilledema across all adjusted models, an association that persisted after adjustment for demographic and cohort variables, although reader identity and cohort membership could not be fully disentangled in this design. Its association with headache was weaker, driven largely by between-cohort differences. Papilledema was recorded as a binary clinical finding without standardized Frisén-scale grading or optical coherence tomography confirmation. These exploratory findings suggest the Evans index may warrant further prospective study beyond NPH, rather than establishing it as a validated marker of intracranial pressure.

## Introduction

Headache and visual disturbance are among the most common indications for cranial magnetic resonance imaging (MRI) in emergency and outpatient neurology and ophthalmology practice. While the primary goal of imaging is to exclude a structural mass lesion, radiologists routinely report two additional metrics on every cranial MRI: the Fazekas score, a semiquantitative grading of periventricular and deep white matter hyperintensity burden [1,2], and the Evans index, the ratio of the maximal frontal horn width to the maximal internal skull diameter, historically used as a surrogate marker of ventricular enlargement [3].

The Evans index was first proposed in the pneumoencephalographic era as a linear surrogate for ventricular volume [4,5] and remains the most widely used linear index for diagnosing ventriculomegaly, with a threshold of >0.3 incorporated into international normal pressure hydrocephalus (NPH) guidelines [6,7]. Volumetric studies show that a single axial ratio captures only part of the three-dimensional change in ventricular geometry, prompting complementary markers such as the callosal angle [8], age- and sex-stratified cut-offs [9], and composite scoring systems such as the iNPH-radscale [10]; the Evans index is therefore best interpreted as one component of a broader radiological picture rather than a stand-alone threshold.

Notably, papilledema is classically considered atypical for NPH, whose diagnostic triad--gait disturbance, cognitive decline, and urinary incontinence--is expected with normal cerebrospinal fluid (CSF) opening pressure [7]. The Fazekas score, in turn, is primarily used to grade age-related small vessel ischemic disease and has been most consistently linked to cognitive and functional outcomes rather than signs of raised intracranial pressure: higher Fazekas grades have been associated with greater risk of cognitive impairment and frailty in prospective aging cohorts [11]. Mechanistically, white matter hyperintensities reflect demyelination, gliosis, and incomplete ischemic injury related to chronic small vessel disease, a pathway distinct from the acute or subacute rise in intracranial pressure that produces papilledema [12].

Papilledema, when present, is a clinically important sign of elevated intracranial pressure and mandates neuroimaging to exclude a mass lesion, venous sinus pathology, or idiopathic intracranial hypertension (IIH) [13], conventionally graded using the Frisén ophthalmoscopic scale despite recognized interobserver variability [14]. IIH predominantly affects obese women of reproductive age, with an incidence far exceeding that of the general population and rising with the global obesity epidemic [15,16]; obesity-driven increases in intra-abdominal and intrathoracic pressure are thought to impair cerebral venous outflow and CSF absorption, providing a plausible link between adiposity and elevated intracranial pressure independent of any structural NPH-type process [17]. Cranial MRI can also show direct orbital correlates of papilledema (optic nerve head flattening, perioptic subarachnoid space distension, optic nerve tortuosity), but these are assessed qualitatively; the Evans index and Fazekas score, by contrast, are measured on sequences already obtained in every routine cranial MRI and offer a more readily available, if less specific, correlate.

Because most headaches are benign and self-limited, current appropriateness criteria recommend neuroimaging only when a red-flag feature is present, such as a new or markedly changed headache pattern, onset after age 50, an abnormal neurological examination, or optic disc oedema [18]. In practice, however, a substantial proportion of patients referred for headache or visual disturbance still undergo cranial MRI, and the Evans index and Fazekas score are reported as incidental findings on nearly every such study. Whether these two frequently reported metrics carry any independent association with clinically documented headache and papilledema, outside the classic NPH or leukoaraiosis literature, has not been well studied.

The aim of this exploratory study was to evaluate, in a cohort of patients undergoing cranial MRI for headache and/or visual disturbance, whether the Evans index and Fazekas score were associated with clinically confirmed headache severity and papilledema, independent of patient age and sex.

## Materials and methods

Study design and population

This retrospective, single-center study included 224 patients who underwent cranial MRI at a tertiary training and research hospital between January 2024 and December 2025 for headache and/or visual disturbance. The study was conducted in accordance with the Declaration of Helsinki and approved by the Istanbul Medipol University Faculty of Medicine Institutional Review Board (IRB: E-10840098-50.04-5711; approval date: 13-08-2026). The population comprised two independent, non-overlapping cohorts, each evaluated by one of two radiologists with five years of post-fellowship experience (Reader A, n=100; Reader B, n=124). Because each reader evaluated a distinct patient group rather than re-reading a shared set of cases, reader identity and cohort membership are, by design, the same variable; this has direct implications for the sensitivity analysis described in the "Statistical analysis" section and is discussed further in the "Discussion" section.

Inclusion Criteria

(i) Age ≥18 years; (ii) cranial MRI performed for headache and/or visual disturbance; (iii) a documented fundoscopic result (papilledema present/absent) and headache severity assessment in the electronic health record, obtained within a clinically appropriate interval of the MRI; (iv) diagnostic-quality MRI without significant motion artifact.

Exclusion Criteria

(i) Prior neurosurgical intervention (e.g., ventriculoperitoneal shunt, craniotomy) or ventricular catheter; (ii) known intracranial mass lesion, hydrocephalus of defined structural cause, cerebral venous sinus thrombosis, or other acute intracranial pathology on the same MRI; (iii) prior diagnosis of idiopathic NPH; (iv) congenital or developmental ventricular anomaly; (v) incomplete documentation of headache severity or fundoscopic status; (vi) non-diagnostic image quality precluding reliable Fazekas or Evans index measurement.

**Figure 1:**
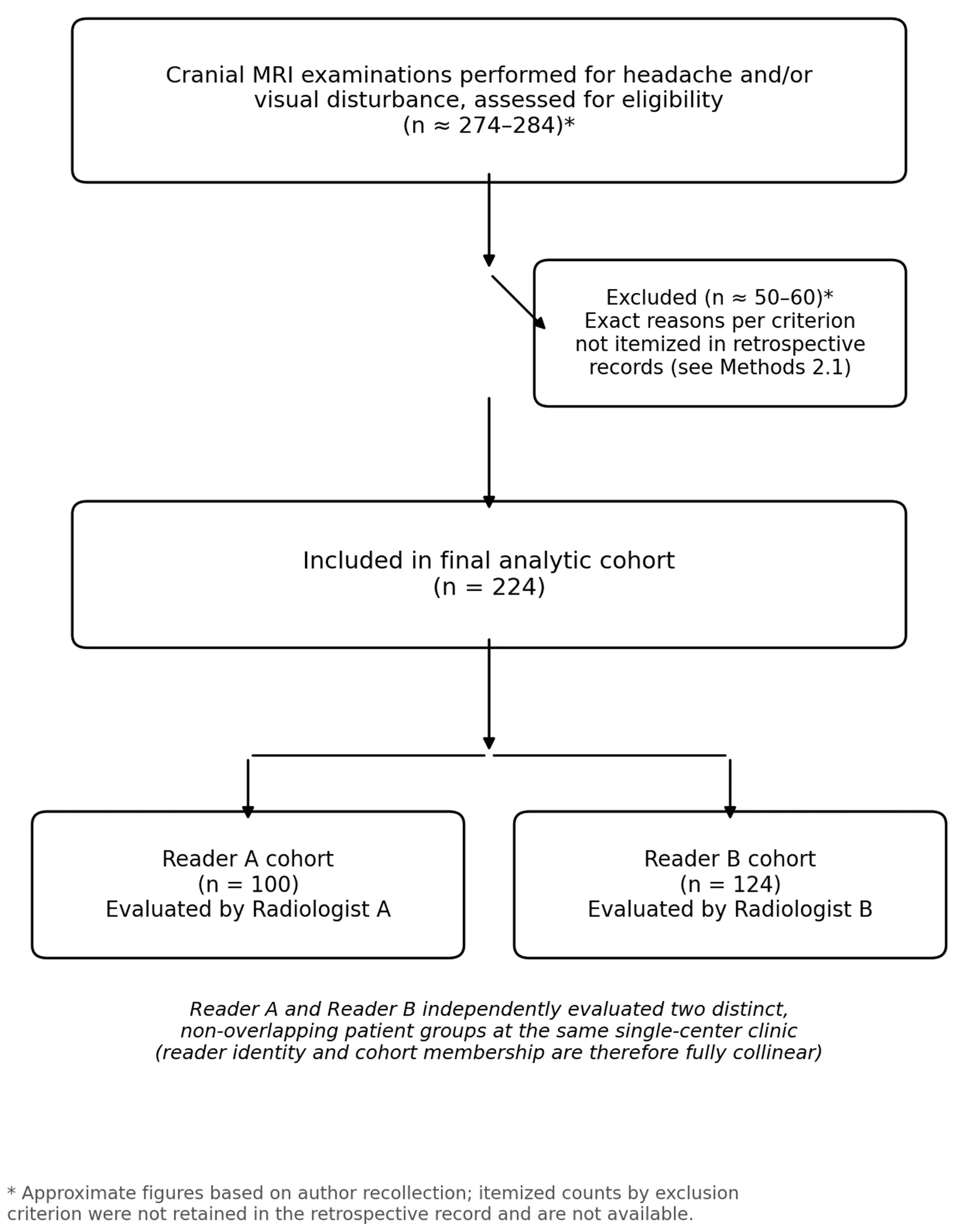
STROBE-style patient flow diagram. STROBE: Strengthening the Reporting of Observational Studies in Epidemiology.

The numbers assessed for eligibility and excluded are approximate figures based on the author's recollection rather than an itemized retrospective log, and exclusions could not be broken down by individual criterion; this is discussed further in the "Study limitations" section (Figure 1).

Imaging protocol

All examinations were performed on a 1.5 T MRI scanner (MAGNETOM, Siemens Healthineers, Erlangen, Germany) using a dedicated head coil, according to the standard institutional protocol: sagittal T1-weighted spin-echo (repetition time (TR)/echo time (TE) ≈ 500/10 ms; slice thickness 5 mm, gap 1.5 mm); axial T2-weighted turbo spin-echo (TR/TE ≈ 4000/100 ms; 5 mm); axial T2 fluid-attenuated inversion recovery (FLAIR) (TR/TE/inversion time (TI) ≈ 9000/120/2500 ms; 5 mm); axial diffusion-weighted imaging (b=0 and 1000 s/mm²; 5 mm); and coronal T2-weighted turbo spin-echo (TR/TE ≈ 4500/100 ms; 4 mm). The field of view was approximately 220-230 mm, and the matrix was approximately 320 × 256. Images were reviewed on a PACS workstation in axial, coronal, and sagittal planes.

Image analysis

**Figure 2:**
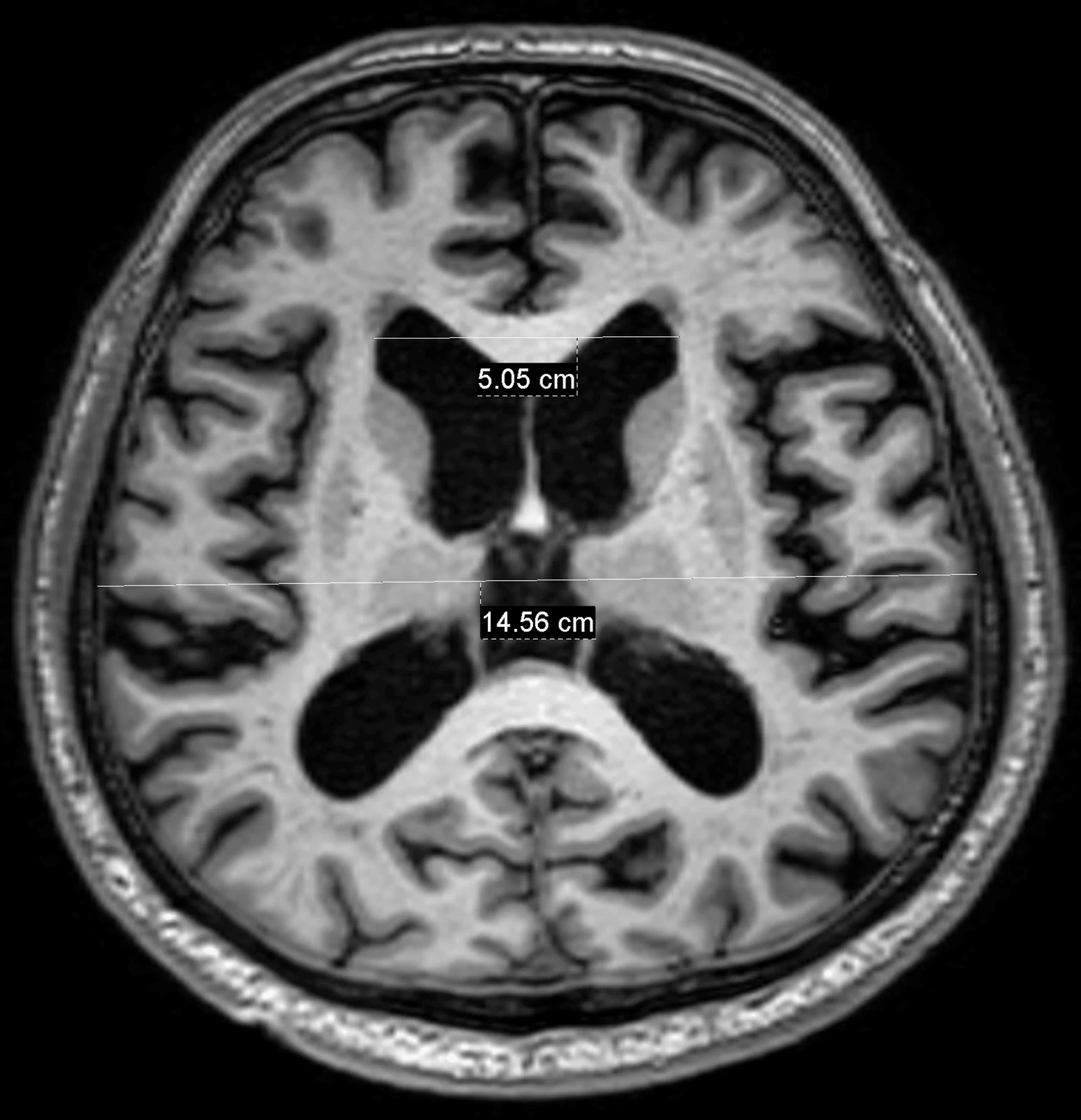
Measurements of the Evans index in the T1W sequence show an increase in the index compared to normal (0.35).

**Figure 3:**
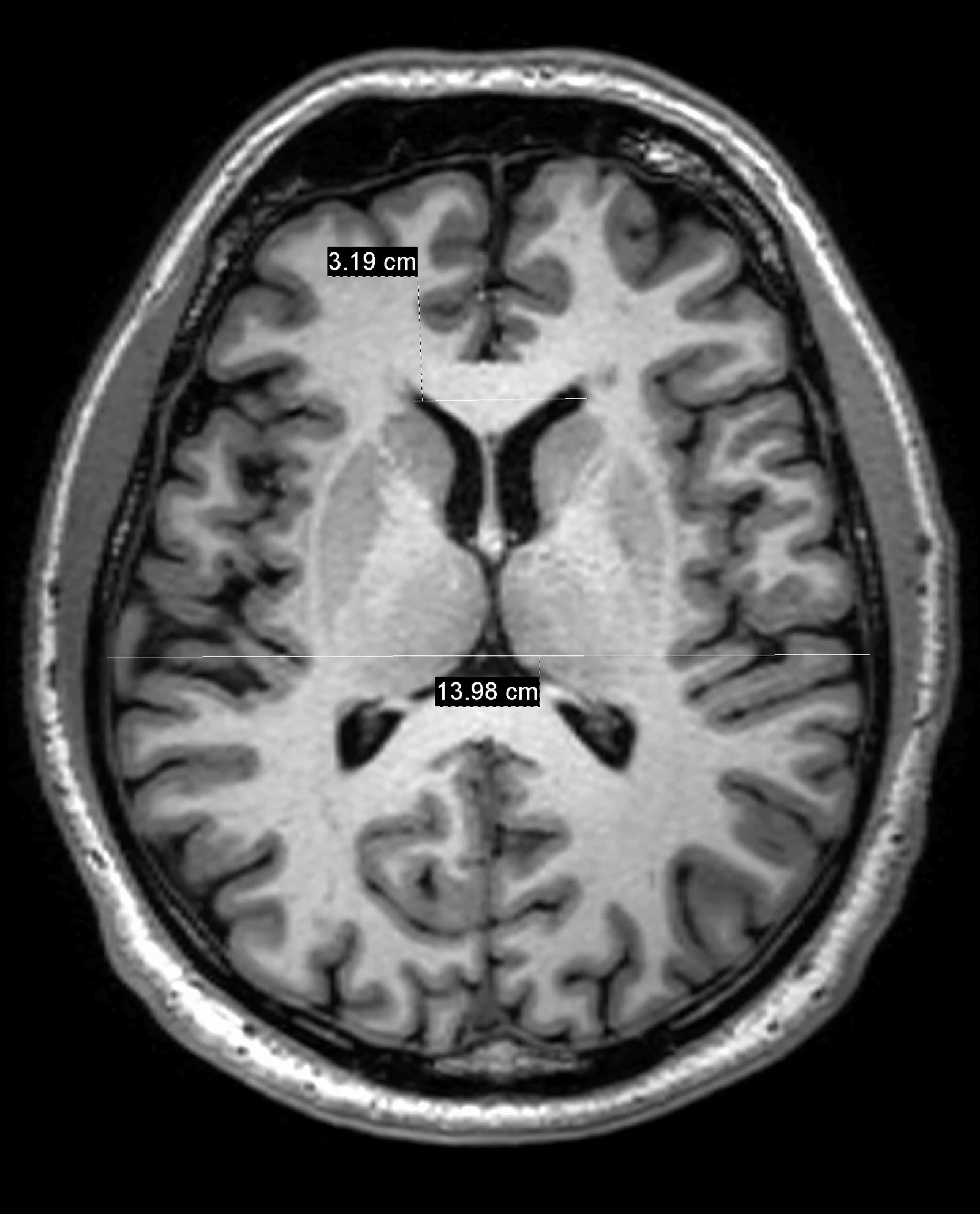
Measurements of the Evans index in the T1W sequence show an index measurement within normal limits (0.23).

**Figure 4:**
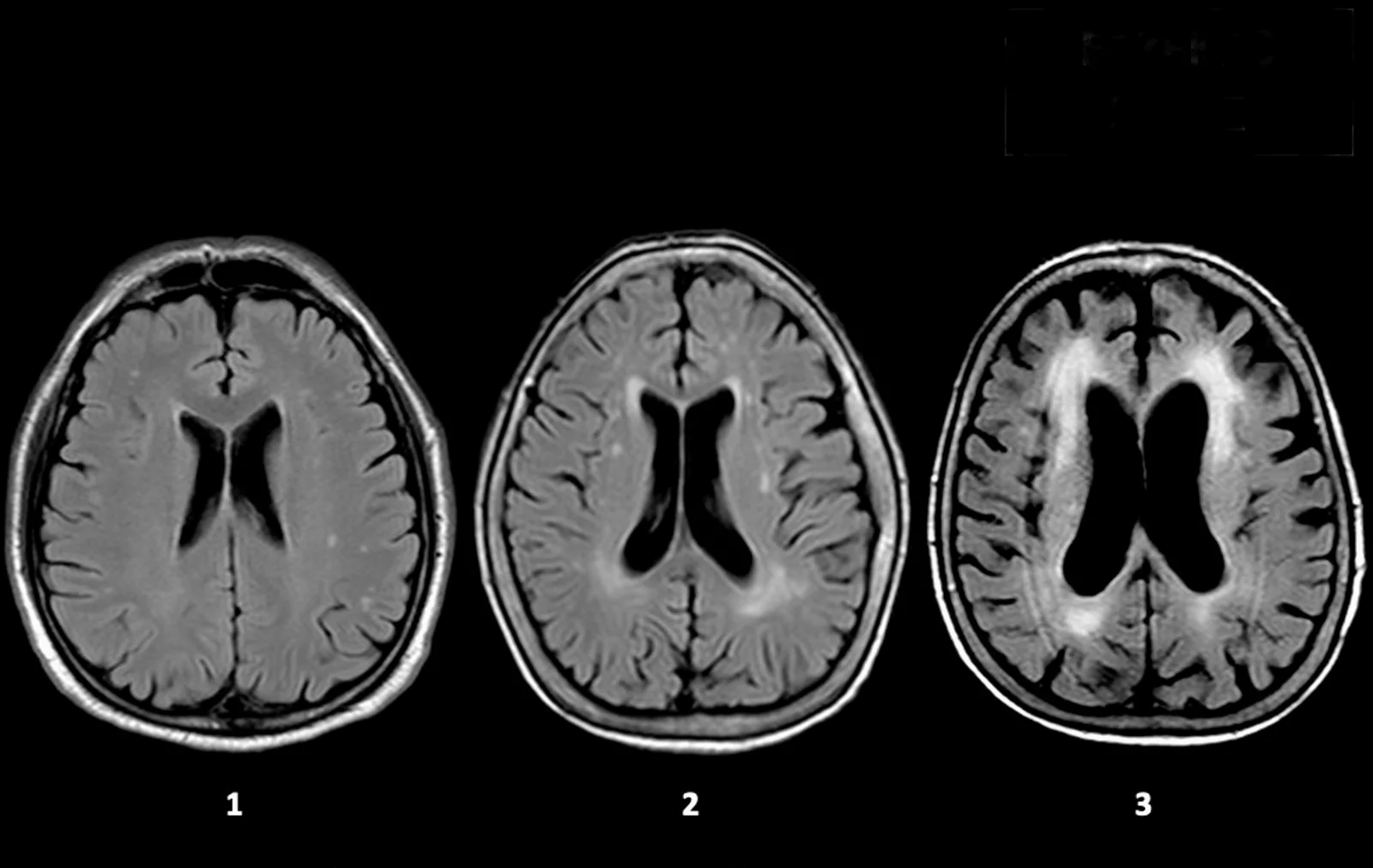
Fazekas score was performed on the T2-FLAIR sequence, and gliotic lesions show a tendency to coalesce, from 1 to 3. FLAIR: fluid-attenuated inversion recovery.

Each examination was independently evaluated by one of the two radiologists, who recorded the Fazekas score (grade 1-3, based on periventricular and deep white matter hyperintensity on FLAIR) (1) and the Evans index (ratio of maximal frontal horn width to maximal internal skull diameter), measured on T1-weighted axial images at the level demonstrating maximal frontal horn width, per the original description and consistent with Figures 2, 3; Fazekas grading was performed on T2 FLAIR images (Figure 4). Further protocol details--whether images were reformatted to a standard reference plane, electronic caliper precision, prior reader calibration, and intra-reader reliability--were not systematically recorded and are noted as a limitation (Figures 2-4).

Readers were blinded to clinical data, including headache severity and fundoscopic findings. Because the two readers evaluated entirely separate cohorts, no shared subset of cases was available for a formal inter-reader agreement (kappa) analysis; this limits the ability to distinguish true biological variation between cohorts from systematic measurement differences between readers, as addressed further in the Discussion.

Clinical data

Headache severity (0=none, 1=mild, 2=moderate, 3=severe) and papilledema (0=absent, 1=present, based on fundoscopic examination) were retrospectively retrieved from the electronic health record, independent of the MRI report. Papilledema was recorded as a binary clinical finding as documented by the examining clinician; a formal Frisén-scale grade was not systematically available [14].

We reviewed the hospital electronic record system for this revision: the identity and specialty of the assessing clinician(s) were not attached to the papilledema finding in the chart; whether headache grading was recorded prospectively or reconstructed retrospectively could not be determined, and the method used to distinguish papilledema from pseudopapilledema or other causes of disc swelling was not documented. These are addressed as limitations below.

Statistical analysis

Continuous variables were expressed as mean ± standard deviation and compared using the Mann-Whitney U test; categorical variables as n (%) and compared using the chi-square test. Key proportions are additionally reported with Wilson 95% confidence intervals. Correlations were assessed with Spearman's rank correlation coefficient. Multivariable logistic regression evaluated the independent association of the Fazekas score and Evans index with (i) papilledema and (ii) headache, adjusting for age and sex. Odds ratios (OR) for the Evans index are reported per 0.05-unit increase for clinical interpretability.

Since Reader A and Reader B evaluated two distinct, non-overlapping cohorts that differed significantly at the outset, reader identity and cohort membership are perfectly collinear. The sensitivity analysis in the "Sensitivity analysis: adjustment for reader/cohort assignment" section, which includes "reader/cohort" as an additional covariate, should be understood not as an independent test of inter-reader reliability, but rather as a re-partitioning of the same between-group heterogeneity; this analysis cannot distinguish the true biological difference between the two patient groups from systematic differences in measurement or interpretation between the readers.

No correction for multiple comparisons was applied across the outcome models, univariate tests, and sensitivity analyses; given the number of statistical tests on a single dataset, reported p-values should be interpreted as hypothesis-generating rather than confirmatory. With 47 papilledema events and 4-5 covariates in the primary model (≈9-12 events per variable), and given the modest sample size, effect estimates and confidence intervals should be interpreted with caution, and reproduction in an independent, adequately powered cohort is needed before any diagnostic threshold is proposed. A two-tailed p<0.05 was considered nominally significant. Analyses were performed using Python (version 3.10, PSF, Oregon, USA) with SciPy (1.10.0) and statsmodels (0.13.5). No formal a priori power analysis was conducted, as this was a retrospective study with a fixed, already-collected sample size.

## Results

The overall cohort (n=224) had a mean age of 70.4 ± 10.6 years (range 47-99) and comprised 120 women (53.6%, 95% CI 47.0-60.0%) and 104 men (46.4%). Fazekas grade 1 was the most common finding (n=139, 62.1%, 95% CI 55.5-68.2%), followed by grade 2 (n=48, 21.4%, 95% CI 16.6-27.3%) and grade 3 (n=37, 16.5%, 95% CI 12.2-21.9%); no patient had a Fazekas grade of 0; whether this reflects the predominantly older age of this population or a coding/selection artifact could not be determined from the available data, and this uncertainty is discussed further in the Limitations. The narrow observed range should be kept in mind when generalizing the Fazekas-related null findings to younger populations.

The mean Evans index was 0.280 ± 0.036. Headache was reported by 121 patients (54.0%, 95% CI 47.5-60.4%; mild in 70, moderate in 45, severe in 6), and papilledema was present in 47 patients (21.0%, 95% CI 16.2-26.8%).

Because inclusion required headache and/or visual disturbance, the “no headache” group (n=103) comprises patients referred primarily for visual disturbance without a co-occurring headache complaint at presentation; the exact breakdown of patients referred for headache alone, visual disturbance alone, or both was not retained and is noted as a limitation.

The Reader A and Reader B cohorts differed significantly in age, Evans index, headache severity distribution, and papilledema frequency (Table 1), consistent with these being two distinct, non-overlapping patient subgroups rather than paired readings of the same patients; accordingly, no inter-rater reliability statistics were calculated, and all subsequent analyses were performed on the pooled cohort.

**Table 1:** Demographic and imaging characteristics of the study population. Continuous variables were compared with the Mann-Whitney U test; categorical variables were compared with the chi-square test. 95% CIs for proportions calculated using the Wilson score method. *p<0.05, Reader A vs. Reader B cohort.

Characteristic	Overall (n=224)	Reader A (n=100)	Reader B (n=124)	p-value
Age, years (mean ± SD)	70.4 ± 10.6	67.1 ± 10.0	73.1 ± 10.3	<0.001*
Sex, female/male, n (%)	120 (53.6)/104 (46.4)	59 (59.0)/41 (41.0)	61 (49.2)/63 (50.8)	0.184
Fazekas grade 1, n (%)	139 (62.1)	70 (70.0)	69 (55.6)	
Fazekas grade 2, n (%)	48 (21.4)	19 (19.0)	29 (23.4)	
Fazekas grade 3, n (%)	37 (16.5)	11 (11.0)	26 (21.0)	0.059
Evans index (mean ± SD)	0.280 ± 0.036	0.267 ± 0.034	0.290 ± 0.036	<0.001*
Headache, none, n (%)	103 (46.0)	55 (55.0)	48 (38.7)	
Headache, mild, n (%)	70 (31.3)	30 (30.0)	40 (32.3)	
Headache, moderate, n (%)	45 (20.1)	13 (13.0)	32 (25.8)	
Headache, severe, n (%)	6 (2.7)	2 (2.0)	4 (3.2)	0.044*
Papilledema, present, n (%)	47 (21.0)	28 (28.0)	19 (15.3)	0.031*

Because reader and cohort are the same variable here, these baseline differences cannot be attributed to measurement disagreement versus true differences between the two patient groups each radiologist happened to be assigned within the same single-center clinic.

Univariate associations

On univariate analysis, the Fazekas grade showed no association with either papilledema (p=0.995) or headache (p=0.666). The Evans index was significantly higher in patients with headache than without (0.284 vs. 0.275, p=0.047), but the difference for papilledema did not reach significance (p=0.206). This discrepancy between the null univariate finding and the significant multivariable finding for papilledema ("Multivariable analysis" section) is consistent with confounding by age: age was positively correlated with the Evans index (ρ=0.291) but, as shown below, was independently associated with lower odds of papilledema, so an unadjusted comparison can mask an association that becomes apparent once age is held constant. Both the Evans index and Fazekas grade correlated weakly to moderately with age (ρ=0.291 and ρ=0.316, respectively; both p<0.001) and with each other (ρ=0.227, p<0.001) (Table 2).

**Table 2:** Univariate associations between imaging and clinical findings (n=224). *p<0.05.

Association (n=224)	Statistic	Test	p-value
Fazekas grade vs. papilledema	χ²=0.01	Chi-square	0.995
Fazekas grade vs. headache	χ²=0.81	Chi-square	0.666
Evans index vs. papilledema	U=3660	Mann-Whitney U	0.206
Evans index vs. headache	U=5270.5	Mann-Whitney U	0.047*
Evans index vs. Fazekas grade	ρ=0.227	Spearman	<0.001*
Age vs. Fazekas grade	ρ=0.316	Spearman	<0.001*
Age vs. Evans index	ρ=0.291	Spearman	<0.001*

Multivariable analysis

After adjustment for age, sex, and Fazekas grade, the Evans index remained independently associated with papilledema (adjusted OR 2.07 per 0.05-unit increase, 95% CI 1.27-3.33, p=0.004) (Table 3), and headache presence (adjusted OR 1.52, 95% CI 1.01-2.25, p=0.047) (Table 4).

**Table 3:** Multivariable logistic regression for papilledema. *p<0.05.

Predictor (papilledema)	Adjusted OR	95% CI	p-value
Fazekas grade (per grade)	1.01	0.63-1.61	0.971
Evans index (per 0.05)	2.07	1.27-3.33	0.004*
Age (per year)	0.97	0.93-1.00	0.040*
Sex (male vs. female)	0.78	0.40-1.54	0.475

**Table 4:** Multivariable logistic regression for headache (presence vs. absence). OR: odds ratio; CI: confidence interval. *p<0.05.

Predictor (headache)	Adjusted OR	95% CI	p-value
Fazekas grade (per grade)	1.09	0.75-1.59	0.656
Evans index (per 0.05)	1.52	1.01-2.25	0.047*
Age (per year)	1.00	0.97-1.03	0.863
Sex (male vs. female)	0.53	0.31-0.92	0.024*

The Fazekas grade was not independently associated with either outcome. Older age was independently associated with lower odds of papilledema (adjusted OR 0.97 per year, 95% CI 0.93-1.00, p=0.040), and male sex was independently associated with lower odds of headache (adjusted OR 0.53 vs. female, 95% CI 0.31-0.92, p=0.024).

Sensitivity analysis: Adjustment for reader/cohort assignment

Because the Reader A and Reader B cohorts differed systematically at baseline (Table 1), a sensitivity analysis added reader/cohort assignment (Reader B vs. Reader A) as an additional covariate to both multivariable models. As noted in the "Statistical analysis" section, reader and cohort are the same variable in this dataset, so this analysis does not test inter-reader reliability independent of true between-cohort differences; rather, it asks whether the Evans index-outcome associations survive once baseline heterogeneity between the two patient groups is statistically accounted for.

The association between the Evans index and papilledema not only persisted but strengthened after this adjustment (adjusted OR 2.76 per 0.05-unit increase, 95% CI 1.59-4.80, p<0.001) (Table 5).

**Table 5:** Sensitivity analysis: multivariable logistic regression for papilledema, adjusted for reader/cohort. *p<0.05.

Predictor (papilledema)	Adjusted OR	95% CI	p-value
Fazekas grade (per grade)	1.03	0.64-1.67	0.894
Evans index (per 0.05)	2.76	1.59-4.80	<0.001*
Age (per year)	0.97	0.94-1.01	0.122
Sex (male vs. female)	0.79	0.40-1.59	0.515
Reader/cohort (B vs. A)	0.31	0.14-0.66	0.003*

Reader/cohort assignment itself was an independent predictor of papilledema (adjusted OR 0.31 for Reader B vs. Reader A, 95% CI 0.14-0.66, p=0.003), and age was no longer independently associated with papilledema once cohort was accounted for (p=0.122). By contrast, the association between the Evans index and headache presence was substantially attenuated and no longer statistically significant (adjusted OR 1.37, 95% CI 0.89-2.09, p=0.148) (Table 6), while reader/cohort assignment itself emerged as an independent predictor of headache (adjusted OR 1.90 for Reader B vs. Reader A, 95% CI 1.06-3.43, p=0.032).

**Table 6:** Sensitivity analysis: multivariable logistic regression for headache, adjusted for reader/cohort. Reader/cohort coded as Reader B vs. Reader A (reference). *p<0.05.

Predictor (headache)	Adjusted OR	95% CI	p-value
Fazekas grade (per grade)	1.08	0.73-1.58	0.700
Evans index (per 0.05)	1.37	0.89-2.09	0.148
Age (per year)	0.99	0.96-1.02	0.539
Sex (male vs. female)	0.51	0.29-0.89	0.017*
Reader/cohort (B vs. A)	1.90	1.06-3.43	0.032*

This pattern is consistent with the originally observed Evans index-headache association being driven, at least in part, by systematic differences between the two cohorts, whereas the Evans index-papilledema association persisted after this adjustment. However, because reader identity and cohort membership are fully collinear in this design, this adjustment cannot demonstrate that the papilledema association is free of measurement or referral differences between the two readers--only that it survives inclusion of the combined reader/cohort indicator in the model.

## Discussion

In this cohort of patients undergoing cranial MRI for headache and/or visual disturbance, the Evans index--but not the Fazekas score--was independently associated with clinically documented papilledema, an association that persisted across adjustment for age, sex, and the combined reader/cohort indicator ("Sensitivity analysis: adjustment for reader/cohort assignment" section), although this adjustment cannot exclude residual confounding or reader-related measurement differences, since reader identity and cohort membership are fully collinear in this design. Its association with headache was weaker and did not survive this adjustment, suggesting the papilledema finding is the more reliable of the two. This is notable because the Evans index is most commonly discussed in the context of NPH, whose diagnostic criteria classically exclude papilledema and assume normal CSF opening pressure [6,7]. An independent statistical association between a higher Evans index and papilledema is one possible pattern consistent with ventricular enlargement secondary to elevated intracranial pressure of another cause (e.g., obstructive or communicating hydrocephalus, or IIH) rather than classic idiopathic NPH; this interpretation is offered as a hypothesis consistent with the observed statistical pattern, and the Evans index itself is a validated measure of ventricular size, not a validated marker of intracranial pressure. The present data did not include CSF pressure measurements, MR venography, or dedicated IIH-specific imaging, and cannot establish this mechanism [19].

The discrepancy between the null univariate association (p=0.206) and the significant multivariable association (p=0.004) for papilledema warrants comment. Age was positively correlated with the Evans index but independently associated with lower odds of papilledema; when two oppositely signed relationships coexist, an unadjusted comparison can obscure an association that becomes visible once the confounder is held constant--a classic suppression pattern. This makes the age-adjusted estimate more informative, but also means the finding should be replicated before being treated as established.

This interpretation is consistent with the broader literature questioning the specificity of the Evans index as an isolated marker of NPH-type ventriculomegaly. Volumetric studies show the index can vary considerably with the exact axial level of measurement and correlates only moderately with true ventricular volume, prompting complementary three-dimensional and structural indices [5,20]. Age- and sex-adjusted reference ranges indicate that a substantial proportion of healthy older adults exceed the traditional 0.3 threshold from age-related brain volume loss alone, without any clinical syndrome [9,21]. Our finding that a higher Evans index tracked with an objective marker of raised intracranial pressure, rather than a gait-cognition-incontinence triad, reinforces the view that the index behaves as a general, non-specific marker of ventricular size whose meaning depends on clinical context--a point also emphasized by studies proposing the callosal angle and composite scales as adjuncts precisely because the Evans index alone cannot distinguish between causes of ventricular enlargement [8,10].

The lack of an independent association between the Fazekas score and either clinical finding is consistent with its primary role in grading chronic small-vessel ischemic change, which is mechanistically distinct from acute or subacute elevation of intracranial pressure [1,12]. Even where the Fazekas score predicts meaningful outcomes, these are almost invariably cognitive, functional, or frailty-related, not related to papilledema or headache [11]. The moderate correlation between the Evans index, Fazekas score, and age most plausibly reflects the shared influence of age-related brain volume loss (ex-vacuo ventricular enlargement); we caution against inferring a causal link between white matter disease and raised intracranial pressure from this correlation, since both markers are well-established correlates of cerebral aging that co-occur without necessarily sharing a causal pathway.

The inverse association between age and the odds of papilledema is consistent with prior observations that papilledema is less frequently identified in older patients, possibly reflecting age-related optic nerve sheath fibrosis limiting transmission of elevated CSF pressure, or greater brain atrophy providing more intracranial compliance before pressure rises sufficiently to produce papilledema. Neither mechanism was directly tested here, and this association should be regarded as requiring independent mechanistic confirmation.

IIH is the paradigmatic condition linking papilledema to a non-NPH cause of raised intracranial pressure and is worth considering whenever papilledema and ventriculomegaly coexist without a classic NPH syndrome [13]. However, IIH is overwhelmingly a disease of obese women of reproductive age (15,16), whereas the present cohort had a mean age over 70 years and a near-even sex distribution (53.6% female); without body mass index data, we cannot determine what proportion of the observed papilledema, if any, was obesity-related.

Several alternative, non-mutually exclusive explanations for the population-level association can be hypothesized, including age-related arachnoid granulation dysfunction, chronic cerebral venous insufficiency, or unrecognized subtle obstructive/communicating hydrocephalus, but none were directly assessed here and should be regarded as candidate hypotheses for future work. Taken together, the absence of papilledema in an older patient with radiological ventriculomegaly should not be taken as reassurance against elevated intracranial pressure, just as papilledema in a younger patient with a modestly enlarged Evans index should prompt consideration of IIH or another secondary cause rather than NPH; both remain clinical hypotheses to be tested prospectively rather than conclusions drawn directly from this retrospective, single-center dataset.

From a practical radiology standpoint, current appropriateness criteria restrict routine neuroimaging in headache to patients with red-flag features--new-onset headache after age 50, a change in headache pattern, an abnormal neurological examination, or documented optic disc oedema--precisely because imaging yield in stable, typical migraine is low [18]. Our patients, by inclusion criteria, had already undergone MRI for headache and/or visual disturbance with documented fundoscopic and headache assessments, so our findings apply to a population in whom imaging was already clinically indicated. Whether radiologists reporting the Evans index as an incidental finding should consider flagging a higher index for clinical correlation when the referral indication includes headache or visual disturbance remains an open question; we do not put this forward as a validated reporting recommendation on the basis of the present, exploratory dataset, and offer it only as a hypothesis for future prospective evaluation.

Study limitations

This study has several important limitations that affect how strongly its conclusions should be weighted.

Reader-Cohort Collinearity

The two reader cohorts were two distinct, non-overlapping patient groups that differed significantly in baseline characteristics rather than the same patients evaluated twice; reader identity and cohort membership are therefore fully collinear. This precluded any interobserver reliability analysis and means the “reader/cohort” sensitivity analysis in the "Sensitivity analysis: adjustment for reader/cohort assignment" section cannot separate a true measurement difference between the two radiologists from a true difference between the underlying populations--it can only show whether the Evans index associations survive after this combined heterogeneity is accounted for. The significant differences in age and papilledema prevalence between cohorts suggest underlying referral or scheduling patterns within the same single-center clinic (rather than distinct outpatient clinics, as clarified above), but this cannot be confirmed from the data available. This is arguably the single most important constraint on interpreting the reported associations and should be resolved through a paired-reader design on a shared subset of cases.

The study was conducted at a single center: the two cohorts reflect two different radiologists at the same clinic independently evaluating two distinct, non-overlapping patient groups, rather than two separate clinical sites.

Statistical Reporting

No correction for multiple comparisons was applied across the univariate tests, the two primary multivariable models, and the sensitivity analyses; reported p-values should be read as hypothesis-generating rather than confirmatory. The papilledema model, with 47 events across 4-5 covariates (≈9-12 events per variable), also has a comparatively low events-per-variable ratio, which can inflate the variance of estimated odds ratios; the relatively wide confidence intervals (Tables 3, 5) reflect this.

Additional statistical diagnostics recommended by the reviewers--complete regression coefficients and standard errors, multicollinearity diagnostics (variance inflation factor (VIF)), formal linearity checks for continuous predictors, influential-observation review, and a penalized (Firth) or bootstrapped sensitivity analysis given the low event-per-variable ratio--were not performed in the present analysis and are noted as a limitation.

Design and Generalizability

The retrospective, single-center design and use of a 1.5 T scanner limit generalizability to other settings and field strengths.

Outcome Measurement

Papilledema was recorded as a binary finding without standardized grading (e.g., modified Frisén scale [14]) or optical coherence tomography confirmation, and headache severity was based on retrospective chart documentation rather than a validated instrument, so some misclassification at category margins cannot be excluded. Only six patients had severe headache, limiting precision of any severity-stratified inference.

The observed papilledema prevalence (47/224, 21.0%) was directly recomputed from the analytic dataset and confirmed to be accurate, ruling out a transcription or coding artifact. This prevalence is nonetheless unexpectedly high for a cohort with a mean age of 70.4 years, particularly after excluding mass lesions, venous sinus thrombosis, structural hydrocephalus, and other acute intracranial pathology. We reviewed the hospital electronic record system specifically for this revision: papilledema status was entered into the patient chart without attribution to a specific examining specialty, and OCT/optic disc photography availability and the final clinical diagnoses of the 47 papilledema patients were likewise not documented in a retrievable, structured form, so the clinical (as opposed to numerical) basis for this prevalence could not be independently verified.

Missing Mechanistic and Risk-Factor Data

Secondary MRI signs specific to IIH (optic nerve sheath distension, posterior globe flattening, empty sella, transverse sinus stenosis) and CSF opening pressure data were unavailable, precluding definitive etiological attribution. Body mass index and several other clinically relevant confounders--hypertension and diabetes status, medication exposure, history of migraine or secondary headache, chronic kidney disease and anemia, sleep apnea, cerebral atrophy, venous sinus abnormalities, and final clinical diagnosis or referral pathway--were also not systematically captured, and residual confounding or differential referral between the two cohorts cannot be excluded as explanations for the observed association [16,17].

Index Selection

We did not employ complementary or three-dimensional ventricular indices such as the callosal angle or a structured composite radscale, which may have provided additional discriminatory information [8,10].

Scope of Analysis

This study characterized statistical associations rather than formal diagnostic performance; sensitivity, specificity, and ROC analyses were beyond the scope. The cross-sectional design does not permit causal inference, and unmeasured confounders related to the underlying reason for ventriculomegaly cannot be excluded.

Reporting Completeness

A STROBE-style patient flow diagram is now provided (Figure 1, "Study design and population" section). The numbers assessed for eligibility and excluded are approximate, based on the authors' recollection rather than an itemized retrospective log, and exclusions could not be broken down by individual criterion; this limits full independent evaluation of selection bias.

Despite these limitations, the findings suggest that the Evans index may retain relevance as an incidental cranial MRI marker beyond the NPH setting, and that its presence alongside headache or papilledema could reasonably prompt consideration of causes of elevated intracranial pressure other than idiopathic NPH--a hypothesis that arises from, but is not definitively confirmed by, the present analysis. Future prospective studies, with a paired-reader agreement analysis, quantitative ventricular volumetry, dedicated IIH-specific imaging assessment, systematic body mass index recording, standardized Frisén-scale grading, an a priori multiple-comparison correction plan, and a larger, adequately powered sample, are warranted to confirm and extend these findings.

## Conclusions

In this retrospective, hypothesis-generating study of patients undergoing cranial MRI for headache and visual disturbance, a higher Evans index was independently associated with clinically documented papilledema, and this association persisted across adjustment for demographic variables and for the collinear reader/cohort variable. An initial association with headache was weaker and appeared to be driven substantially by between-cohort differences that could not be separated from between-reader differences in this design. The Fazekas score showed no association with either clinical sign. These findings suggest, but do not by themselves establish, that ventricular enlargement on cranial MRI in this demographic should not be reflexively attributed to idiopathic normal pressure hydrocephalus. Whether radiologists should flag an elevated Evans index for clinical correlation with papilledema is a hypothesis raised by this exploratory analysis, not a recommendation supported by it; prospective, adequately powered studies with a paired-reader design are needed before this observation could be translated into a reporting recommendation.
